# The genetic variation of different developmental stages of *Schistosoma japonicum*: do the distribution in snails and pairing preference benefit the transmission?

**DOI:** 10.1186/s13071-020-04240-w

**Published:** 2020-07-20

**Authors:** Meng-Jie Gu, Yan-Wei Li, Aidan M. Emery, Shi-Zhu Li, Yong-Zhong Jiang, Hui-Fen Dong, Qin-Ping Zhao

**Affiliations:** 1 Department of Parasitology, School of Basic Medical Sciences, Wuhan University, Wuhan, 430071 Hubei China; 2 Hubei Provincial Center for Disease Control and Prevention, Wuhan, 430072 Hubei China; 3 Department of Life Sciences, Natural History Museum, Cromwell Road, London, SW7 5BD UK; 4 National Institute of Parasitic Diseases, Chinese Center for Disease Control and Prevention, National Center for Tropical Diseases Research, WHO Collaborating Center for Tropical Diseases, Key Laboratory of Parasite and Vector Biology, Ministry of Health, Shanghai, 200025 China

**Keywords:** *Schistosoma japonicum*, Genetic variation, Cercaria, Adult worm, Miracidium, Distribution, Pairing

## Abstract

**Background:**

*Schistosoma japonicum* is a waterborne parasite that causes schistosomiasis in humans and in more than 40 animal species. *Schistosoma japonicum* shows distinct genetic differentiation among geographical populations and multiple hosts, but the genetic diversity of different developmental stages of *S. japonicum* from is less studied. Such studies could elucidate ecological mechanisms in disease transmission by analysing feedbacks in individual physiology and population state.

**Methods:**

After infection using cercariae from a pool of snails shedding together (Method I) and infection using mixed equal numbers of cercariae from individually shed snails (Method II), different developmental stages of *S. japonicum* were genotyped with microsatellite loci, including 346 cercariae, 701 adult worms and 393 miracidia. Genetic diversity and molecular variation were calculated at different population levels. Kinships (*I′*) among cercariae at intra-snail and inter-snail levels were evaluated. Genetic distance (*Dsw*) was compared between paired and unpaired worms, and partner changing was investigated through paternity identification for miracidia.

**Results:**

The cercaria clones in individual snails varied from 1 to 8 and the kinship of cercariae within individual snails was significant higher (*P* < 0.001) than that among different snails after deleting near-identical multi-locus genotypes (niMLGs). The allelic diversity of worms in Method I was lower (*P* < 0.001) than that in Method II, and allele frequency among mice in Method I was also less consistent. The parents of some miracidia were worms that were not paired when collected. The *Dsw* between each female of paired and unpaired males was much larger (*P* < 0.001) than that between the female and male in each pair.

**Conclusions:**

Most of the infected snails contained multiple miracidia clones. The aggregation of genetically similar *S. japonicum* miracidia in individual snails and the unbalanced distribution of miracidia among snails suggests a non-uniform genetic distribution of cercariae among snails in the field. This further influenced the genetic structure of adult worms from infections with different cercariae sampling methods. *Schistosoma japonicum* in mice can change paired partner, preferring to mate with genetically similar worms. These characteristics provide implications for understanding the balance in genetic diversity of *S. japonicum* related to the transmission of schistosomiasis.
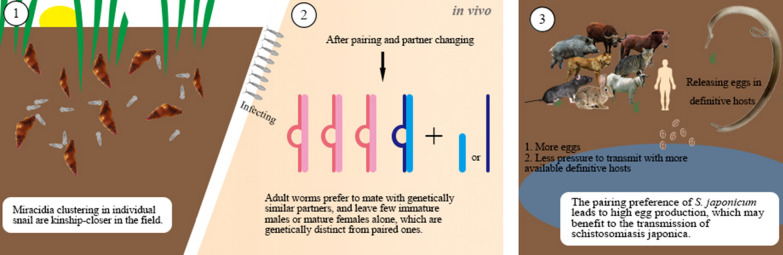

## Background

Schistosomiasis is one of the most prevalent parasitic diseases and causes severe socio-economic losses and public health problems worldwide [1]. Although the infection rate of schistosomiasis in humans and buffaloes has decreased dramatically [2], the distribution area of the intermediate snail host, *Oncomelania hupensis*, remains large in China, with only a recent 5.6% decrease over 10 years [2, 3]. The re-emergence of *O. hupensis* snails and schistosomiasis patients in some transmission interrupted areas [4] and anthropogenic changes such as water conservancy projects, expansion of wetland area, climate change, population migration [5] may lead to expansion of endemic areas. Some anthropogenic changes could influence the genetic diversity and population structure of *Schistosoma japonicum* [5–8]. Geographical differentiation among *S. japonicum* populations in China has been observed using a variety of markers, including nuclear genes [9, 10], mitochondrial genes [11, 12], microsatellite loci [13, 14], and genome-wide single nucleotide polymorphisms (SNPs) [15, 16]. The population differentiation of *S. japonicum* was thought to result from geographical separation, habitat isolation [12, 17], co-evolution with different species of snails [18–20], and different transmission patterns driven by definitive hosts [17, 21, 22]. All of these studies focused on the genetic diversity of *S. japonicum* among geographical populations or among different species of hosts. The genetic mechanism on biology is less studied both at the individual physiological level and at the population level, including the changed distribution pattern and genetic structure of *Schistosoma* during different developmental stages in the life-cycle, which may highlight important ecological mechanisms driving schistosomiasis.

Miracidia invade specific species of snails and develop into sporocysts that eventually generate cercariae, which are shed out into water to seek and penetrate a compatible host, leading to human schistosomiasis infection. *Biomphalaria glabrata* snails could be infected with multiple miracidia, previously implied by the detection of multiple genotypes of *S. mansoni* cercariae shed from individual snails [23, 24]. From one to nine *S. japonicum* genotypes have been detected from a single *O. hupensis* snail when ten cercariae were genotyped [25]. However, many genotypes of cercariae in an individual snail could be identified as near-identical multi-locus genotypes (niMLGs) caused by somatic mutation during the asexual amplification of miracidium to cercariae, rather than multiple infections [26], which makes it difficult to determine the true burden of miracidial penetration for snails in the field, and complicates the distribution pattern and population structure of *Schistosoma* in snails.

There are more than 40 domestic and wild mammals that serve as definitive hosts of *S. japonicum* [22, 27, 28]. The recovery rate of adult worms varies from different species of definitive hosts infected with the same pool of cercariae [29]. It is hard to clarify whether this difference is the result of selective pressure from hosts or genetic variation among cercariae. The divergence could be enlarged between the founder population (adult worms) and the original population (cercariae) by the founder effect in the extreme case [30]. Wang et al. [22] considered that *S. japonicum* from water buffalo, cattle, and humans were differentiated significantly from those from goats, pigs, dogs, and cats. Rudge et al. [17] found limited differentiation of *S. japonicum* among these hosts. This inconsistency may mainly relate to the genetic differences originating from cercariae in different snails redistributed by flood and limited sample size, in addition to the selective pressure under different definitive hosts. Since it is challenging to collect adult worms from naturally infected hosts in the field, artificial infection of laboratory hosts has been essential, especially for studies in population genetics, immunology, and pharmacology. The different methods of sampling or infection, including cercariae release or collection from snails and infection using different cercariae populations, may affect the genetic distribution of the parasite in definitive hosts.

Adult worms of *S. japonicum* are located mainly in the portal and mesenteric veins of definitive hosts. Worms show genetic differentiation among groups from the liver, hepatic-portal-vein, and mesenteric blood vessels [31]. This diversity led us to investigate the genetic differentiation between eggs trapped in the liver and excreted through the intestine. Eggs in the liver and eggs excreted through the intestine play different roles in pathogenicity and transmission of *S. japonicum*, respectively. In addition, the pairing of adult worms is a prerequisite for egg production and contributes to the genetic diversity of eggs. Partner changing has been reported for schistosomes, for example, *S. mansoni* females were more likely to switch mates when the newcomer male was more dissimilar to themselves than their first mate [32], and hetero-specific pairs of worms from co-infection of *S. mansoni* and *S. intercalatum* tend to change partners and revert to homo-specific pairing [33, 34]. Then, how is the genetical similarity and heterozygosity of *S. japonicum* which is homo-specific mating?

In this study, the genetic variation of *S. japonicum* at different life stages was investigated, including cercariae from snail hosts and adult worms and eggs from definitive hosts. Cercariae were further investigated by identifying the number of genotypes per individual snail (hence the number of miracidia originally infecting it) and by estimating kinship of cercariae within and among snails. After mice were infected with two methods that collected cercariae either by pooled snail shedding or mixing equal numbers of cercariae from each snail shed individually, the genetic structure of the adult worm population from the mice was compared with the original cercarial population, and the consistency in allelic distribution of adult worms among mice was evaluated. Furthermore, the phenomenon of partner changing of *S. japonicum* in mice was confirmed, and pairing preference of adult worms was investigated with genetic distance between worms.

## Methods

### Sample collection

**Fig. 1:**
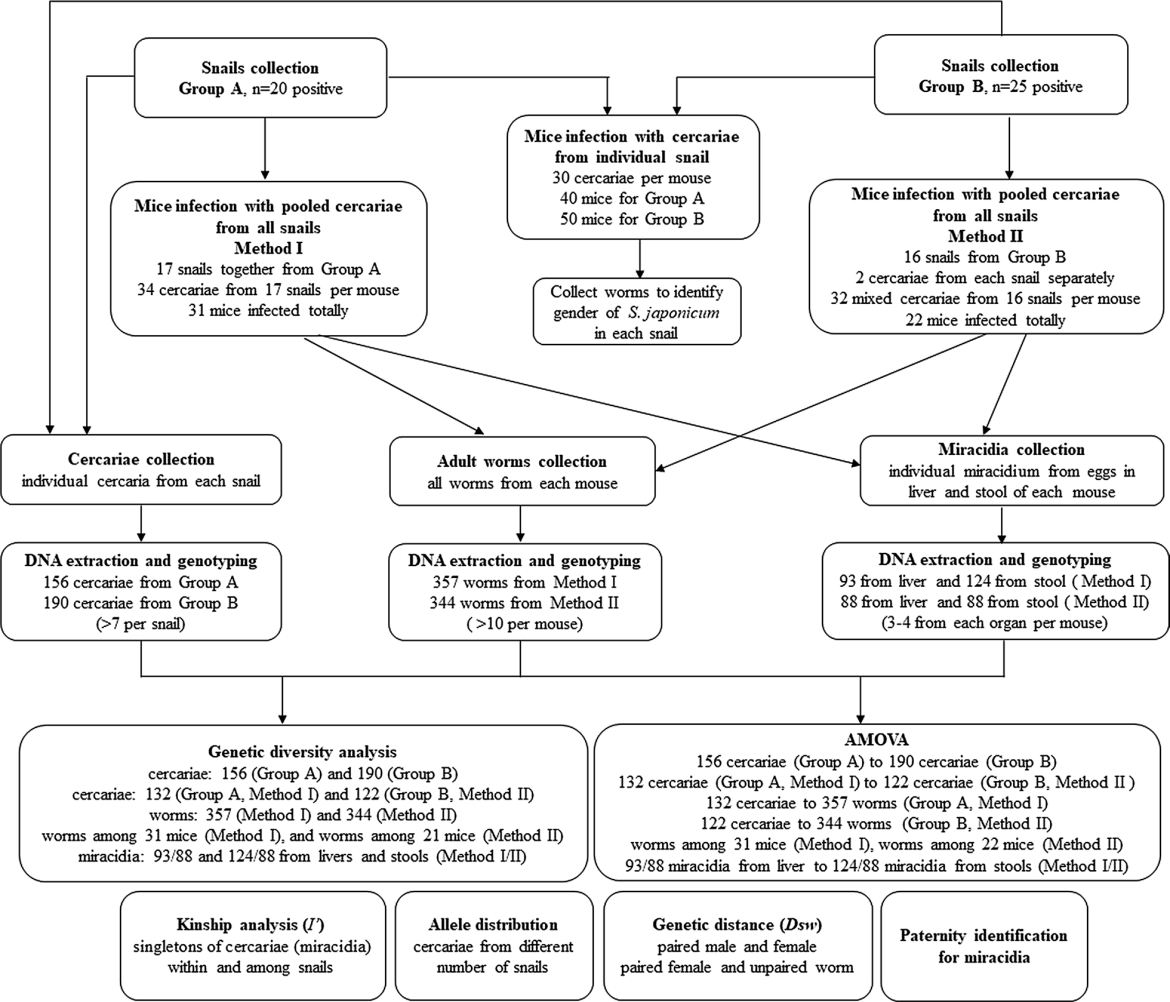
Flowchart of sample collection and experimental design

Sample collection and experimental design are summarised in Fig. 1. The sample collection steps are outlined below, including snail collection, cercariae collection, mice infection and adult worm collection, and miracidia collection.

### Snail collection

*Oncomelania hupensis* snails were collected in March and September 2016 from the eastern area of Dongting Lake (29° 32′ 5.25″ N, 112° 52′ 35.00″ E), Hunan province, China. Snails were located in ditches in 3 villages at a distance of 10 km. The collection site is a marshland and not a national park or other protected area or private land. Collections were carried out with the permission and assistance of the Institute of Schistosomiasis Control of Hunan Province, China, which supervises snail control in the area. *Oncomelania hupensis* is not an endangered or protected species. Snails were brought back to the laboratory and classified as Group A for those collected in March and Group B for those collected in September. After a month of captivity, snails were washed and transferred to individual water-filled vials for 3 h under light at 25 °C, to stimulate the emergence of cercariae for identifying *S. japonicum* infection. Finally, 20 snails from Group A and 25 snails from Group B were identified as infected with *S. japonicum*.

#### Cercariae collection

After cercariae were released from individual snails, some were transferred to a dish using a loop and washed in cercariae washing solution (5 g/l lactalbumin hydrolysate, 6.8 g/l NaCl, 0.4 g/l KCL, 0.2 g/l CaCl_2_, 0.2 g/l MgSO_4_·7H_2_O, and 1 g/l glucose) under microscopy. After 3 washes, cercariae were pipetted individually with 2 μl sterilized washing solution onto a Whatman™ FTA card (GE Healthcare, Pittsburgh, USA). After drying, cards were stored in a sealed plastic bag in a desiccator at room temperature. At least 50 cercariae per snail were collected individually and stored onto Whatman™ FTA cards.

#### Mice infection with cercariae from individual snail

To distinguish single-sex *S. japonicum* infected snails, 2 laboratory Kunming mice were infected percutaneously with 30 cercariae from each snail per mouse. In total, 40 mice were infected for 20 snails of Group A, and 50 mice were infected for 25 snails of Group B. Worms were retrieved from the mesenteric veins of each mouse by perfusion using 0.9% NaCl and dissection 7 weeks later after infection. Care was taken to minimise the potential of splitting paired adult worms caused by mechanical force during perfusion and dissection, using 3 experienced technicians to process the worms as quickly as possible. Each pair of worms were transferred to separate tubes for washing and marked immediately, and were stored in absolute ethanol in pairs once separating spontaneously. Unpaired worms from each mouse were checked under microscopy to confirm the sex and then stored in ethanol.

#### Mice infection with cercariae pooled from all snails (Method I and II) and adult worm collection

For subsequent infections, 17 of the 20 snails from Group A and 16 of the 25 from Group B remained alive and able to produce sufficient cercariae. Two different cercariae pooling methods were used for the two groups of snails. For the 17 remaining snails of Group A, all snails were pooled together in a flask containing water to stimulate the emergence of cercariae (Method I). Thirty-one mice were then infected percutaneously with 34 cercariae for each mouse. For the infection using 16 snails of Group B, 2 cercariae from each snail shedding individually were pooled together, and the mixed 32 cercariae were used to infect a mouse (Method II), replicating to infect 22 mice in total. All mice were sacrificed to collect *S. japonicum* worms 7 weeks post-infection as described above. Finally, 530 worms from mice by Method I (Group A), 484 worms from mice by Method II (Group B) were collected.

#### Miracidia collection

Before all infected mice were sacrificed to collect adult worms, stools from each mouse were collected for 7 days, to concentrate eggs excreted through the intestine for miracidia hatching. Egg sediment from each mouse was exposed to dechlorinated water in a flask under light at 25 °C to hatch miracidia. Miracidia were harvested from the upper water of each flask by a pipette and transferred into a dish with miracidium washing solution (5 g/l lactalbumin hydrolysate, 6.5 g/l NaCl, 0.14 g/l KCl, 0.12 g/l CaCl_2_, and 0.20 g/l NaHCO_3_). After 3 washes, miracidia were pipetted individually with 2 μl sterilised washing solution onto a Whatman™ FTA card. After being dried, the card was stored in a sealed plastic bag in a desiccator at room temperature. At least 50 miracidia per mouse stool were collected.

Miracidia hatched from eggs trapped in livers of infected mice were also collected as follows. The liver of each infected mouse was collected after adult worm collection, homogenised in cold 1.2% NaCl, and filtered to obtain the egg sediment. Miracidia were hatched using the same method as that used for the stool sample. Finally, at least 50 miracidia per mouse liver were pipetted individually and stored onto Whatman™ FTA cards.

### Genomic DNA extraction

For DNA of a single cercaria or miracidium sample, a 2 mm diameter disc was removed from the centre of each sample pipetting area of a Whatman™ FTA card using a 2 mm Harris Micro-punch (GE Healthcare Life Sciences, Stevenage, UK). The disc was placed in the base of a 96 well, 1.2 ml U-bottom plate. After one wash with 100 μl FTA purification reagent (GE Healthcare Life Sciences) for 5 mins followed by 100 μl TE buffer (10 mM Tris-Cl, 0.1 mM EDTA, pH 8.0), 14 µ of 0.1 M NaOH with 0.3 mM EDTA, pH 13.0 was added to each well and the solution left to incubate for 5 mins, after which 26 µl of 0.1 M Tris-HCl buffer, pH 7.0 was added. Then the plate was mixed by pulsing with a vortex mixer 3 times for approximately 10 s each. The solution was then left at room temperature for 10 min, pulse vortexed 10 times, and the eluate was transferred to a clean 96-well plate, and stored at − 20 °C for further use.

As for individual adult worms, the anterior part, including the uterus, was cut off under microscopy from each adult female worm to avoid the influence of eggs in the uterus on the genotype of the female worm. While the whole male or immature female worms were used for DNA extraction. The total genomic DNA was extracted individually from each worm using a standard sodium dodecyl sulfate-proteinase K procedure [35] as follows. Each worm was incubated and thawed in 400 μl extraction buffer containing 50 mM Tris-HCl, 50 mM EDTA, 100 mM NaCl, 1% SDS, and 100 mg/ml proteinase K, at 56 °C for 1 h with gentle mixing. DNA in solution was extracted using standard phenol/chloroform purification, followed by 3 M sodium acetate (pH 5.2) and ethanol precipitation. Pellets of DNA were washed in 70% ethanol, air-dried, and resuspended in 20 μl TE (pH 8.0), stored at − 20 °C as crude DNA extract of adult worm for further use.

### PCR amplification and microsatellite genotyping

To confirm genomic DNA existed in FTA card elution or crude extract from ethanol stored sample, the *28S* ribosomal DNA gene fragment was amplified from DNA solution of a single cercaria, miracidium, or adult worm, using following primers: forward (5′-GTG GAG TTG AAC TGC AAG C-3′) and reverse (5′-GCT CAA CAW TAA TAG TCA AAC CTG-3′). PCRs were performed using the Illustra PuReTaq Ready-To-Go PCR Beads in a 96-well plate (GE Healthcare Life Sciences, USA). For each bead, 2 µl of FTA elution from single larva or 0.5 µl of crude extract from a single worm were added, with 1 µl of each primer, and water to a final volume of 25 µl. The amplification was implemented with the following program: 95 °C for 5 min; followed by 40 cycles of 30 s at 95 °C, 30 s at 60 °C, and 40 s at 72 °C; and a final extension step at 72 °C for 7 min. Reactions were then checked by running 5 µl on a 2% agarose TAE gel.

Small scale genotyping was done for 30 cercariae, 30 miracidia, and 30 worms which were chosen randomly, to construct an optimal multiplex microsatellite loci panel for PCR amplification. Twenty loci developed and validated in previous studies [26, 36] and from our laboratory were checked. Finally, 9 microsatellite loci (Additional file 1: Table S1) were selected out to form the panel. Microsatellite loci for each DNA sample were genotyped using the Type-it Microsatellite PCR kit (Qiagen, Manchester, UK) with the multiplex of 9 loci. The forward primer of each pair was labelled with an appropriate fluorescent dye, including 6-FAM, HEX, TAMRA, and ROX (Additional file 1: Table S1). PCR reactions were prepared in a 96-well PCR plate using 6.25 µl of 2× master mix, 1.25 μl Q solution, 0.5 μl primers (10 μM), 2 μl DNA template, and 2.5 μl ddH_2_O, then carried out in a Bio-Rad thermocycler with the following program: 95 °C for 5 min; followed by 35 cycles of 30 s at 95 °C, 90 s at 60 °C, and 3 mins at 72 °C; followed by a final extension step at 60 °C for 45 min. PCR reactions were checked by running 5 µl on a 2% TAE agarose gel, and successful amplifications were sent to Sangon Biotech (Shanghai, China) for fragment analysis on an ABI3100 genetic analyzer (Applied Biosystems, Foster City, CA, USA). Genotypes were scored using a microsatellite plugin of Geneious 11.0 [37]. All samples were amplified and genotyped twice to reduce the deviation. In total, genotypes of 346 cercariae, 701 adult worms, and 393 miracidia were generated for the following analyses.

### Genetic diversity analyses

Genetic diversity of each locus was estimated in all genotyped cercariae by calculating the observed number of alleles (*Na*), the effective number of alleles (*Ae*) in GenAlEx 6.5 [38] and allelic richness (*Ar*) and gene diversity (*Hs*) in hierfstat ver. 0.04–22 [39], including 156 cercariae from 20 snails of Group A and 190 cercariae from 25 snails of Group B. Deviation from Hardy-Weinberg equilibrium (HWE) for each locus was tested with the Chi-square test in GenAlEx, and the linkage disequilibrium test between loci was calculated in SHEsis [40].

The *Na*, *Ae*, *Ar* and *Hs* were calculated for different populations during the process of mice infection as follows. For cercarial populations used in infection, 132 cercariae from 17 snails of Group A (used in Method I) and 122 cercariae from 16 snails of Group B (used in Method II) were evaluated. After genotyping and filtering low-quality data, at least 5 pairs of adult worms (when present) and all unpaired worms from each mouse were successfully genotyped. In total, 357 worms from mice in Method I (using cercariae from 17 pooled snails of Group A), 344 worms from mice in Method II (using equally mixed cercariae from 16 individual snails of Group B) were involved. Additionally, the genetic diversity of adult worms in each mouse was estimated to check the allelic consistency of adult worms among mice in Method I and II separately. For miracidium populations, genetic diversity of miracidia from liver and stool was calculated at supra-population level (miracidia from livers or stools of all mice), due to only 3–4 miracidia from liver or stool in each mouse were genotyped, including 93 miracidia from livers (3 per mouse) and 124 miracidia from stools (4 per mouse) in Method I, and 88 miracidia from both of livers and stools (4 per mouse separately) in Method II.

### Analysis of molecular variance (AMOVA)

The genetic differentiation of all genotyped cercariae from Group A and Group B was estimated using AMOVA in Arlequin ver. 3.5.2.2 [41]. After excluding snails not used for mice infections, the differentiation between 132 cercariae from 17 snails in Group A (for Method I) and 122 cercariae from 16 snails in Group B (for Method II) was tested. After infection, the differentiation between 357 adult worms in Method I and the original cercarial population (132 cercariae from group A) was tested. Also, the 344 worms in Method II was tested against the 122 cercariae from group B. In addition, the variation of adult worms among 31 mice in Method I and adult worms among 22 mice in Method II were evaluated separately. For miracidia, the genetic differentiation was tested between 93 miracidia from livers and 124 miracidia from stools in Method I, and the same for 88 miracidia from both livers and stools in Method II.

### Kinship analysis for singletons of cercariae with niMLGs

The number of cercarial genotypes from each snail was counted. Since it is difficult to distinguish typing errors or somatic mutation during sporocyst multiplication from the original miracidium genotype, a group of cercariae genotypes with fewer than 2 mismatched alleles were identified as niMLGs and assigned to a lineage derived from the same miracidium in each snail. Finally, the number of miracidia that established genetic clones in each snail was evaluated based on the lineage number using the R package *allelematch* ver. 2.5 [42].

Since cercariae with niMLGs in one snail were presumed to be derived from one miracidium [26], one of the genotypes (singleton) for niMLGs was assigned to represent a group of cercariae which originated from a miracidium with the function *amCluster* in allelematch, to produce the filtered dataset for kinship analysis between miracidia. Then, kinship among singletons (miracidia) was estimated using the allele size correlation coefficient (*I′*) according to Streiff [43] in SPAGeDi ver. 1.5a [44] at intra-snail and inter-snail levels for 20 snails in Group A and 25 snails in Group B, to find out whether aggregation of miracidia in one snail correlated with the genetic similarity of miracidia.

### Allele distribution for cercaria subsets from the different number of snails

The non-uniform genetic distribution of cercariae among snails implies that sample size of snail hosts would affect the allele distribution of the cercaria suprapopulation (cercariae from all snails). *Na*, the basic index for indicating gene abundance of a population [45, 46], was further evaluated for each locus in different cercariae subsets by selecting different numbers of snails randomly. Snails were selected randomly at each given number of snails, which was set from 1 to maximum in each group using the base function sample in R, with 100 replicates. Under each replicate, the number of cercariae in each snail was set to 5, which was determined according to the practical feasibility and the average number of cercariae genotyped in each snail in this study. The genotypes of cercariae for the same number of snails were merged, and the *Na* was then estimated for each locus with the function *nb.alleles* in R package *hierfstat*. The relativity between genetic diversity and snail number was evaluated by calculating the smallest sample size of snails when 50% and 95% of total *Na* was set.

### Genetic distance between female and male adult worms

To detect a potential genetic preference for *S. japonicum* during mating, the inter-individual genetic distance *Dsw* [47] between the male and female of each pair in individual mice was measured with Populations ver. 1.3.32 (http://www.bioinformatics.org/~tryphon/populations/), and compared with that between the paired female one and unpaired worms (if available, unpaired is mostly male) in the same host.

### Paternity identification for miracidia

The allele frequencies of all adult worms and miracidia in each mouse were analysed and calculated using the *Allele Frequency Analysis* function in Cervus 3.0 [48]. All 9 loci were finally assessed as suitable markers for further parentage analysis with a combined non-exclusion probability of less than 0.01%. The function *Simulation of Parentage Analysis* was applied based on allele frequency, to estimate the resolving power of codominant loci and critical values of the log-likelihood (LOD) with assumptions as follows: 10,000 offspring (miracidia) for each individual mouse; 10 candidate mothers and fathers in each mouse (estimated sample size according to the number of adult worms collected in a host); the proportion of candidates was set to 100% of genotyped adult worms; other options were set by default. Finally, both of the outputs from the *Allele Frequency Analysis* and *Simulation of Parentage Analysis* were referred in the *Parentage Analysis* module, to assign each offspring to its most likely parents. The parents that have more than 2 alleles mismatched with offspring were excluded, and those that had the biggest LOD were considered most likely as the parents.

### Statistical analysis

All data used in statistical analysis in this study are continuous data. Significance of difference on genetic diversity indices (*Na*, *Ae*, *Ar* and *Hs*) of loci, kinship coefficient (*I′*), and inter-individual genetic distance (*Dsw*) between two groups were evaluated with the significance level of 0.05. First, the normal distribution of datasets was checked with Sharpiro-Wilk test. Paired-samples/independent t-test was then applied when datasets met normal distribution. Otherwise, the Wilcoxon signed-rank test and Mann-Whitney U-test were applied for the related/independent samples. Bonferroni correction was used to correct the *P*-value in multiple comparisons.

## Results

### Genetic diversity and differentiation of *S. japonicum* within and among different groups

#### Cercarial populations

All loci in 346 cercariae met the linkage equilibrium (D′ < 0.7, *r*^2^ < 0.3), and deviated from the Hardy-Weinberg equilibrium (*P* < 0.001). Nine microsatellite loci showed high polymorphism in cercariae, including 156 cercariae from the 20 snails (7.5 ± 0.5 (mean ± standard deviation) cercariae checked per snail) of Group A and 190 cercariae from the 25 snails (7 ± 1 cercariae checked per snail) of Group B. Allele number (*Na*), the effective number of alleles (*Ae*), allelic richness (*Ar*), and gene diversity (*Hs*) for each locus are given in Additional file 2: Table S2. No significant difference in genetic diversity was observed for all loci of cercariae between Group A and B (*t*_(8)_ = 0.978, *P* = 0.357 for *Na*; *t*_(8)_ = 2.125, *P* = 0.060 for *Ae*; *t*_(8)_ = 1.179, *P* = 0.272 for *Ar*; *Z* = − 1.866, *P* = 0.062 for *Hs*) (Additional file 2: Table S2). AMOVA showed the differentiation of cercariae between Group A and B was 1.89%. This indicated that the genetic difference is very limited between the two cercaria groups.

#### Infection-related populations: cercariae and adult worms

For comparison between populations related to mice infections, cercariae from snails not used for infection were excluded in the genetic diversity analysis and AMOVA. The *Na*, *Ae*, *Ar*, and *Hs* for all 9 loci of 132 cercariae from 17 snails in Group A were 17.0 ± 2.75, 9.3 ± 2.20, 17.0 ± 2.75, and 0.89 ± 0.03, respectively. All indices were higher (*t*_(8)_ = 3.277, *P* = 0.011 for *Na*; *t*_(8)_ = 4.312, *P* = 0.003 for *Ae*; *t*_(8)_ = 3.416, *P* = 0.009 for *Ar*; *Z* = − 2.524, *P* = 0.012 for *Hs*, with corrected *P* < 0.025 as significance) than those for 357 worms from Method I (Additional file 3: Table S3). For 122 cercariae from 16 snails in Group B, *Na*, *Ae*, *Ar*, and *Hs* were 16.0 ± 3.56, 7.8 ± 2.77, 16.0 ± 3.56, and 0.85 ± 0.07. All indices showed no significant difference (*t*_(8)_ = − 1.265, *P* = 0.242 for *Na*; *t*_(8)_ = 1.116, *P* = 0.297 for *Ae*; *t*_(8)_ = − 1.603, *P* = 0.171 for *Ar*; *Z* = − 0.896, *P* = 0.370 for *Hs*) with that for 344 adult worms from Method II (Additional file 3: Table S3). This implies that Method I reduces the genetic diversity of the adult worms compared with the original population of cercariae (132 cercariae), and the worms from Method II maintains a similar genetic diversity with the original cercarial population (122 cercariae). Furthermore, the genetic diversity for all 9 loci of adult worms in each mouse varied considerably among mice in both methods, especially in Method I (Additional file 4: Table S4). The *Na* and *Ar* of adult worms in 22 mice of Method II were both significantly higher than worms in 31 mice of Method I (*U* = 607, *Z* = 4.805, *P* < 0.001 for *Na*; *t*_(51)_ = − 4.034, *P* < 0.001 for *Ae*), which suggested that adult worms in each mouse from Method II contained more alleles than worms of each mouse from Method I.

AMOVA showed limited differentiation as 2.32% between two cercariae groups from snails used to infect mice (132 cercariae of Group A and 122 cercariae of Group B). The differentiation is also limited between the adult worms and the original population of cercariae (1.29% for 357 worms of Method I and 132 cercariae of Group A, 0.75% for 344 worms of Method II and 122 cercariae of Group B). The differentiation of worms among mice were 0.49% and 0% for worms among 31 mice in Method I and worms among 22 mice in Method II, respectively. It revealed that worms among mice in Method II have higher consistency in allelic distribution than worms among mice in Method I.

#### Miracidiual populations

The *Na* and *Ar* of miracidia from stools were similar with that from livers of mice (*Z* = 0.241, *P* = 0.809 for *Na*; *Z* = − 0.415, *P* = 0.678 for *Ar*) in Method I but *Ae* and *Hs* are different (*t*_(8)_ = − 4.650, *P* = 0.02 for *Ae*; *Z* = 2.442, *P* = 0.015 for *Hs*) (see Additional file 5: Table S5). While the *Na*, *Ae*, *Ar*, and *Hs* of miracidia from stools were all similar with that from livers of mice in Method II (*t*_(8)_ = 1.492, *P* = 0.174 for *Na*; *t*_(8)_ = 0.558, *P* = 0.592 for *Ae*; *t*_(8)_ = 1.472, *P* = 0.174 for *Ar*; *t*_(8)_ = 0, *P* = 1.0 for *Hs*). The genetic differentiation is limited between miracidia from livers and stools, with 1.02% in Method I and 0% in Method II.

### Genetic distribution of cercariae in snails

#### Miracidia developed in the individual snails are genetically similar

Of the infected snails, 65% (13 of 20) snails of Group A, and 92% (23 of 25) snails of Group B shed a single-sex of *S. japonicum* that could originate from one or multiple miracidia infection. Among them, the ratio of female- to male-containing snails was 6:7 and 11:12 for the two groups, respectively, showing no parasite sex-ratio bias between snails. The other snails, carrying both sexes of adult worms, must have been infected with more than one miracidium. Furthermore, multi-locus genotypes (MLGs) of cercariae ranged from 6 to 8 (7.75 on average) in 20 individual snails of Group A, and from 5 to 8 (6.65 on average) in 25 individual snails of Group B. After near-identical multi-locus genotypes (niMLGs) were filtered, the number of clones was inferred as from 3 to 8 in individual snails of Group A and from 1 to 6 in individual snails of Group B, with an average of 6.6 and 4.2, respectively. This multi-clonal distribution even after filtration further implied that the multiple infections with more than one miracidium in a snail is common in the field.

**Fig. 2:**
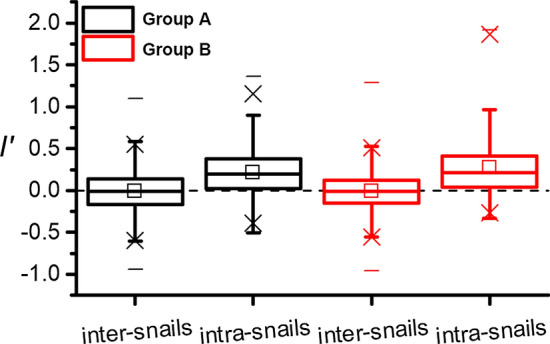
Frequency distribution of kinship coefficients for cercariae inter- and intra-snails. Box plot of pairwise *I’* estimated with niMLGs-filtered data of cercariae inter- and intra-snails of two groups. Minimum and maximum are marked with “-”, the “×” means 99% values included, and the arithmetic mean is represented by a square

After niMLGs were filtered, the allele size correlation coefficient, *I′* of miracidia was 0.01 on average at the inter-snail level in both Group A and B (Fig. 2), which implied that the similarity of genotypes of miracidia among different snails is very limited. While the *I′* of miracidia increased to 0.21 on average (from − 0.50 to 1.36) at intra-snail levels of Group A and increased to 0.27 (from − 0.33 to 1.91) in snails of Group B, both were higher (*t*_(409.4)_ = − 14.649, *P* < 0.001 for Group A; *U* = 765784.5, *Z* = 12.836, *P* < 0.001 for Group B) than that between snails (Fig. 2). This high kinship coefficient of miracidia at intra-snail levels confirmed that miracidia were more genetically similar within snails than that among snails.

#### Sampling efforts affect the genetic diversity of cercariae

**Fig. 3:**
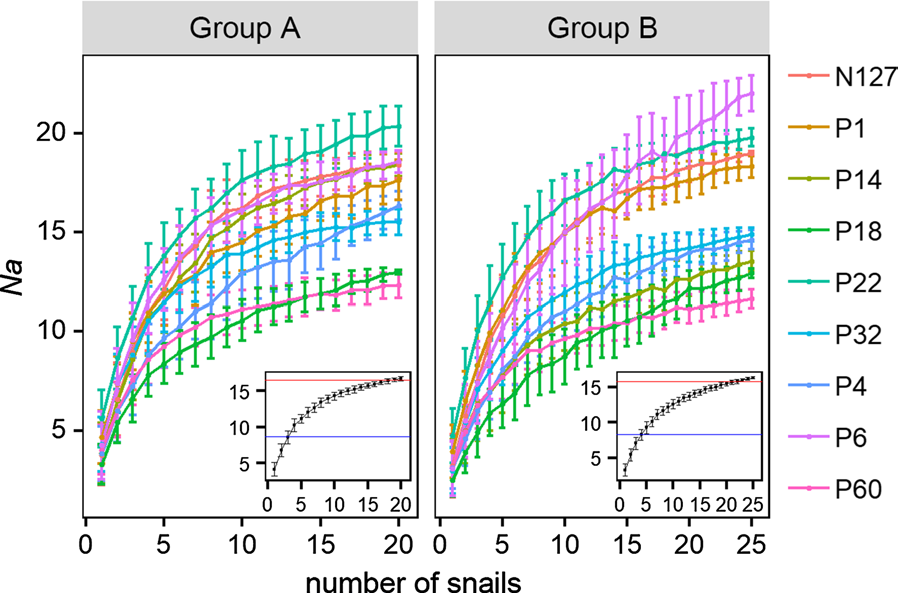
The number of snails sampled affects the coverage of alleles of cercariae. Nine curves represent *Na* of cercariae on 9 loci separately in snails of Group A or B, which increased with more snails involved. Standard deviation of *Na* among replicates for each locus progressively decreased with the number of snails increasing. The blue horizontal line in the inset shows 50% of total *Na*, and the red line shows 95% of total *Na* for all 9 loci. Standard deviation of *Na* among replicates decreases with increasing number of snails

In total, 157 different alleles distributed in cercariae from 20 snails of Group A for 9 loci (17.4 per locus). The deviation of *Na* on all loci is significant among replicates when the number of snails is limited (Fig. 3). More alleles can be covered, and the variation of *Na* among replicates decreased dramatically when the number of snails increased (Fig. 3). The number of snails needs to be 17–19 (85%) in Group A when *Na* of 7 loci (N127, P60, P14, P4, P1, P32 and P6) of cercariae reach the plateau (where *Na* has no significant difference (*P* < 0.05) with the total *Na* of the corresponding locus) separately, while 23 or 24 (92%) snails need to be sampled in Group B when the *Na* of 4 loci (P60, P22, P1 and P32) could reach the plateau. *Na* of other loci could not reach the plateau until all snails were sampled, which implied that 9 loci had varied allelic frequency among snails (Fig. 3). When all loci were considered together, cercariae collected from 4 snails (20%) were enough to retrieve 50% total alleles (8.7 per locus), with a deviation of 8.5%. The number of snails should increase to 19 (95%) when the number of retrieved alleles was set to 95% of the total (16.6 per locus), with the deviation of 1.7%.

Similarly, 150 different alleles were identified for 9 loci of cercariae from 25 snails of Group B, with an average of 16.7 per locus. Five snails (20%) was minimal to retrieve 50% total alleles (8.3 per locus) of cercariae, with a deviation of 9.0%, and 23 snails (92%) was necessary to retrieve 95% total alleles (15.8 per locus), with a deviation of 1.5% (Fig. 3).

### The pairing preference of *S. japonicum* in mice

**Fig. 4:**
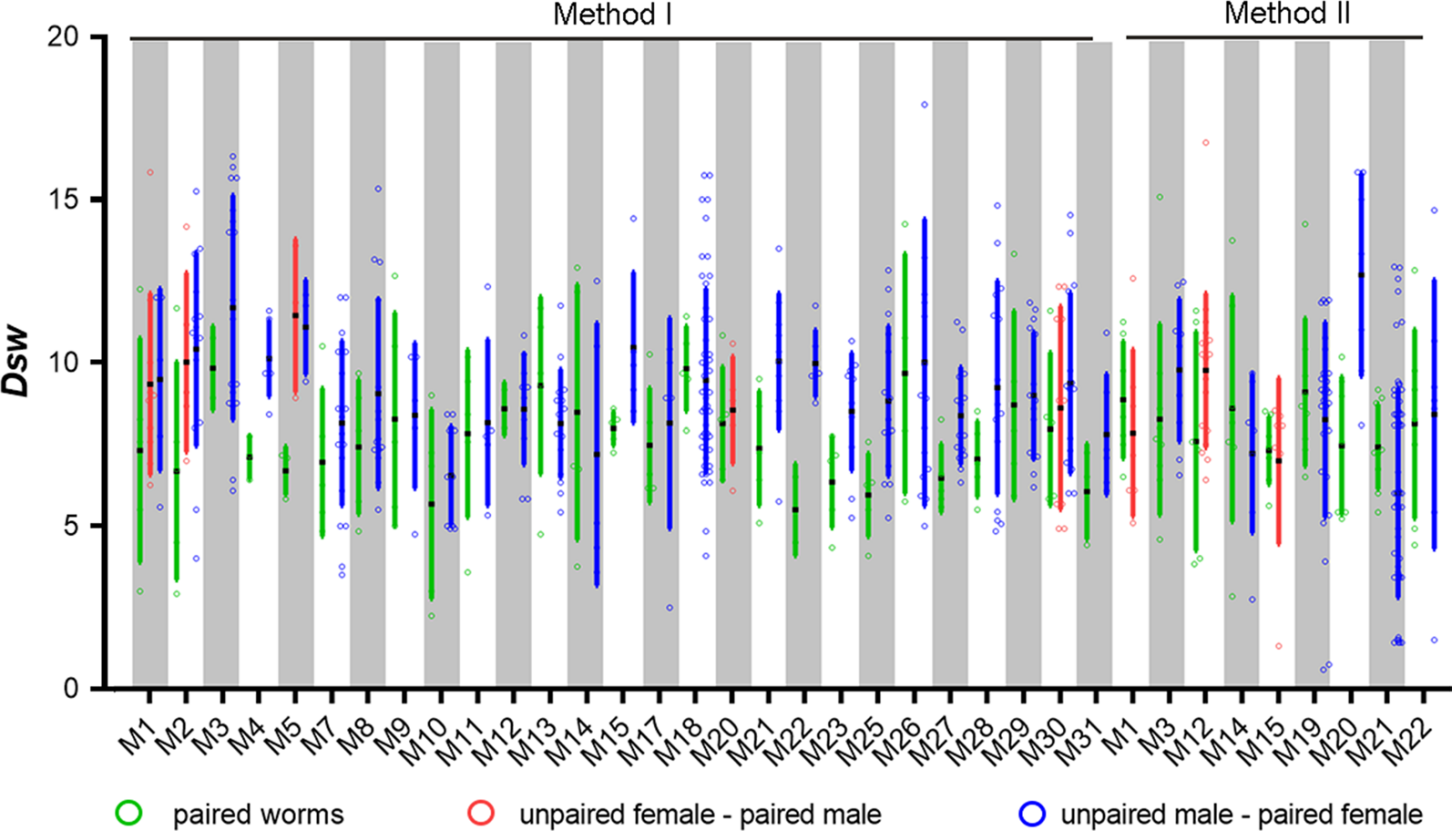
Genetic distance between paired and unpaired worms. Spot plot of genetic distance between each pair of paired worms, between unpaired females (if available) and paired male, and between paired female and unpaired males (if available) in 36 mice which have unpaired worms, marked by green, red, and blue circles separately. Black dots represent the average mean of genetic distance, and error bars are displayed as coloured bars

Among 53 mice, 17 mice carried only paired worms. The other 36 hosts contained paired and unpaired worms, including 4 mice in which all unpaired worms were females, 28 mice in which all unpaired worms were males, and 4 mice in which unpaired worms were both females and males. On average, 13 ± 4.76 worms were genotyped for each mouse. For 8 mice that contained unpaired females, *Dsw* ranged from 1.3 to 16.7 (9.0 ± 2.7) between each paired male and unpaired females in the same host. It showed no significant difference (*t*_(7)_ = − 2.197, *P* = 0.064) with *Dsw* between male and female in each pair (range: 2.9–7.7; mean: 7.7 ± 2.3). While for 32 mice that contained unpaired males, *Dsw* ranged from 1.5 to 20.6 (9.2 ± 2.8) between each paired female and unpaired males in the same host, larger than that between the female and male in each pair (range: 2.3–15.1; mean: 7.7 ± 2.4) (*t*_(32)_ = − 4.435, *P* < 0.001, with a corrected *P* < 0.025 as the significance level) (Fig. 4). This suggests *S. japonicum* might prefer to mate with a genetically similar partner.

Thirty-seven of the genotyped 393 miracidia (9.4%) were successfully assigned to their most likely parents (Table 1), even when most of the collected adult worms were genotyped. When allowed allele mismatch was increased to 4, the rate of parent identification increased to 44.6%, suggesting that allele mismatch may be responsible for the low rate of parent identification for miracidia. Among them, 25 miracidia were offspring of adult worms which were paired when collected, the assigned parents of the other 38 miracidia were not paired with each other when collected, and the father or mother was coupled with a heterosexual partner that was not a parent of the miracidium. For example, miracidia of M28-L-6 and M28-L-7 from Method I were assigned to the same father (M28-m2) but different mothers (M28-f2 and M28-f3). Similarly, M9-L-7 and M9-L-8 from Method II, were from the same mother (M9-f1) but different fathers (M9-m1 and M9-m3). For M16-S-6 from Method II, the assigned father was unpaired, and the assigned mother was pairing with another male. These results provide genetic evidence that *S. japonicum* mates with different partners over time.

**Table 1:** Paternity identification for miracidia

Group	Offspring ID	Mother ID	Father ID
Method I	**M2-S-8**	**M2-f1**	**M2-m5**
	M3-L-7	M3-f1	M3-m1
	**M7-L-5**	**M7-f2**	**M7-Single-m1**^a^
	**M10-S-6**	**M10-f4**	**M10-m1**
	**M19-L-6**	**M19-f3**	**M19-m2**
	**M20-S-6**	**M20-f2**	**M20-m3**
	M24-S-7	M24-f5	M24-m5
	**M27-L-5**	**M27-f5**	**M27-m3**
	**M28-L-6**	**M28-f3**	**M28-m2**
	M28-L-7	M28-f2	M28-m2
	**M30-S-5**	**M30-f4**	**M30-m1**
	**M30-S-8**	**M30-f4**	**M30-m1**
Method II	M1-S-6	M1-f5	M1-m5
	M1-S-7	M1-f1	M1-m1
	M2-S-7	M2-f3	M2-m3
	M2-S-8	M2-f3	M2-m3
	M3-S-7	M3-f1	M3-m1
	M3-L-5	M3-f8	M3-m8
	**M7-L-8**	**M7-f3**	**M7-m2**
	M8-L-7	M8-f1	M8-m1
	**M9-L-7**	**M9-f1**	**M9-m3**
	M9-L-8	M9-f1	M9-m1
	M11-L-5	M11-f7	M11-m7
	M13-L-5	M13-f3	M13-m3
	**M16-S-6**	**M16-f8**	**M16-Single-m1**
	**M16-L-6**	**M16-f4**	**M16-m2**
	**M16-S-8**	**M16-f2/5**	**M16-m3**
	M17-S-8	M17-f5	M17-m5
	M18-S-5	M18-f1	M18-m1
	M19-S-8	M19-f5	M19-m5
	M19-S-8	M19-f6^a^	M19-m6
	M20-L-5	M20-f5	M20-M5
	**M21-S-6**	**M21-f7**	**M21-m6**^a^
	**M21-L-5**	**M21-f5**	**M21-m3**
	M21-L-6	M21-f2	M21-m2
	**M21-L-8**	**M21-f7**^a^	**M21-m6**
	M22-S-8	M22-f5	M22-m5

^a^Indicates there are multiple potential candidates with same LOD

*Notes*: Offspring ID is the identity of miracidium, which is composed of host number, origin organ of sampling, and miracidium number. M, S, and L represent mouse, stool, and liver, respectively. For example, M2-S-8, represents the 8th miracidium that was hatched out from a stool (S) of the 2nd mouse (M2). Mother or father ID assigned is the identity of the adult worm that assigned to be the mother or father of the corresponding miracidium (offspring), includes host number, the sex of the worm, and worm number, f and m represent the female and male, respectively. Single means the worm was not in a pair when collected. M2-f1 means the female one of the 1st worm pair in the 2nd mouse. The parents of miracidia which were not paired with each other at the time of collection are in bold

## Discussion

A total of 45 snails were found to be infected with *S. japonicum* from over 8000 snails collected in the field. Through MLGs evaluation, most of the snails were confirmed to be infected with more than one miracidium of *S. japonicum*, with 6.6 and 4.2 cercariae clones on average per snail in the two groups, respectively. Of 45 snails, 80% (32/45) contained single-sex *S. japonicum*, and most of them were infected with multiple cercaria clones. Only one (3.1%) snail was infected with a single miracidium. This overdispersion is common for parasites within hosts in the field [49]. The unpredictable behaviour of some individuals in the host population [50], the spatial distribution of parasites with infectivity, few hosts in the whole population susceptible to parasites, and low prevalence of parasites [51] have all been confirmed to lead to aggregated distribution of the parasite in the host. In this study, the kinship coefficient (*I′*) derived from miracidium genotypes in individual snails was significantly higher than those among different snails, indicating that miracidia clustering together in the same intermediate snail host have closer kinship than in different snails. This aggregation of genetically similar *S. japonicum* miracidia in individual snails and disequilibrium distribution of miracidia among snails may be explained with the following circumstances: (i) snail immune pressure: miracidia selected by the snail immune system and only those that have compatible alleles (and being more related) can survive when encountering a particular snail genotype; (ii) genetics: miracidia could exclude those with lower genetic similarity through the competition when a snail is multi-infected; (iii) snail control: snail populations were isolated into several subpopulations by strict snail control, which facilitates inbreeding of *S. japonicum* in separate populations, then amplifies the aggregation of *S. japonicum* with closer kinship and separate distribution among snails.

This non-uniform genetic distribution of cercariae among snails further implies that the sample size of snail hosts would affect the genetic diversity of the cercarial suprapopulation. Therefore, the sample size of snails should be evaluated to make sure cercariae from selected snails can cover most of the alleles of *S. japonicum* at the population level. In this study, 20% of snails is enough to retrieve 50% of the total alleles of cercariae. It reflects that many alleles are shared among cercariae in different snails. However, sampling 95% of total alleles is difficult, as approximately 90% of snails were needed to cover the lower-frequency alleles. It is necessary therefore to understand the genetic diversity and distribution of alleles in cercariae among snails, to get the appropriate sample size for cost-effective population studies, especially for regions at low prevalence, where the degree of parasite overdispersion is higher [51], and allele frequencies of parasite subpopulations would be dramatically affected by the population quantity of snails sampled.

The adult worms in mice obtained with two infection methods both showed no significant genetic differentiation from the corresponding original cercarial population in the AMOVA. However, the two methods have a different ability to maintain the genetic diversity of cercariae through the process of infection. The genetic diversity of adult worms in 22 mice from Method II (using equally mixed cercariae from each snail) was similar to that of the original cercarial population, and the genetic diversity of adult worms in 31 mice from Method I (using cercariae from pooled snails) decreased when compared to that of the original cercarial population. Furthermore, the variation of adult worms among mice in Method I was larger than that in Method II. It implies that infection with equally pooled cercariae from individual snails (Method II) transfers more alleles from cercariae to adult worms in each mouse. The allele distribution among mice in Method II was more consistent than that in Method I. It is reasonable that cercariae from different snails in Method I do not have an equal chance to infect a mouse, which creates a larger genetic differentiation of adult worms between mice in Method I than that in Method II. The uneven distribution and release of cercariae on the surface of the water from each snail would make the cercariae sampling replicates more variable from a pool of snails using Method I, thereby altering the allele frequency of the adult worm population. Method II is therefore recommended for artificial infection, which can transfer more alleles from cercariae to adult worms and keep the consistency in the genetic structure of adult worms in mice.

Referring to the genetic difference discovered between adult worms in different organs [31], we suspected that miracidia from eggs deposited in the liver and intestine might possess a different genetic background, which then influences the pathological function of eggs in the liver and the transmission role of eggs in stool. In this study, the genetic diversity was similar between miracidia from liver and stool, except *Ae* and *Hs* in Method I which may result from the unequal sample size (3 *vs* 4), and no significant differentiation was shown. These data indicated that eggs of *S. japonicum* were transferred to the liver or intestine randomly.

Mating is an essential process in the life-cycle of *Schistosoma*. It has been suggested that *S. mansoni* could separate and re-pair to a genetically dissimilar partner, which may increase the adaptive ability of offspring [32]. Only 9.4% miracidia found possible parent pairs in this study, and the rate of parent identification increased to 44.6% when the allowed allele mismatch was increased to 4, suggesting that allele mismatch may be responsible for the low rate of identification. The mutation of microsatellite loci of miracidia during the sexual reproduction of *S. japonicum* may cause deviation from the allele sizes of original adult worms, which can differ among loci [52], thus making parental identification difficult. Regardless, the parents successfully identified for miracidia indicate that partner changing is common for *S. japonicum* adult worms. Previous studies reported that hetero-specific pairs always intend to re-mate to homo-specific pairs in a mixed infection of *S. mansoni* and *S. intercalatum* [33, 53], males were more active in the process of partner changing [33]. This may be the consequence of the male-bias of adult worms. In our study, one female was also occasionally found being embraced by two males after perfusion. For *S. japonicum*, the genetic distance (*Dsw*) between unpaired worms (mainly unpaired males) and paired females, was higher than that between the male and female in each pair. This indicates a tendency for *S. japonicum* to mate with a genetically similar partner, contrary to *S. mansoni*. Huang et al. [54] reported that mating with a genetically dissimilar partner would decrease the production of *S. japonicum* eggs in definitive hosts and hatching rate of eggs. It is known that egg-laying capacity and pathogenicity to definitive hosts of *S. japonicum* is more significant than that of *S. mansoni* [55]. *Schistosoma mansoni* tends to produce less pathogenic eggs and cause less damage to hosts, even with the same number of cercariae as *S. japonicum*. Such adaptations are suitable for continuation of adult worm population of *S. mansoni* in the field, where few mammalian species can be used as definitive hosts. However, *S. japonicum* tends to produce many more eggs, which produce severe pathological damage, accelerating host death, while using more than 40 mammalian hosts. Considering that *S. japonicum* prefers a genetically similar partner to produce more eggs, we speculate that the different preference of pairing between *S. japonicum* and *S. mansoni* is driven by the difference in egg productivity, pathological damage, and available definitive hosts species in the life-cycle. How the distribution of *S. japonicum* in snails and pairing of adult worms affect the balance between adaptation by maximizing genetic diversity and virulence or transmission by minimizing genetic diversity deserves further investigation.

## Conclusions

This study investigated the genetic diversity and kinship of *S. japonicum* miracidia (cercariae filtered niMLGs out) in intermediate snail hosts in the field, the genetic structure of different developmental stages in mice by different sampling methods, and pairing preference of adult worms in mice. We found that infection with multiple miracidia is common in individual snails, with the aggregation of genetically similar miracidia in individual snails and disequilibrium distribution among snails. The sampling method during the infection has an impact on the genetic structure of adult worms. Partner changing and paring with genetically similar adult worms helps to balance the genetic diversity of *S. japonicum* for adaptation and transmission. All of the results give better understanding of the genetic variation of different developmental stages of *S. japonicum*. The genetic distribution characteristics and pairing preference of *S. japonicum* may provide an insight into genotype-related forces that affect the survival and transmission of this species.

## Supplementary information

**Additional file 1: Table S1.** The information for nine microsatellite markers used in this study.

**Additional file 2: Table S2.** Genetic diversity of nine microsatellite loci in all genotyped cercariae from snails of two groups.

**Additional file 3: Table S3.** Genetic diversity of each locus in adult worms from mice infected with two methods.

**Additional file 4: Table S4.** Genetic diversity of combined nine loci in worms from each mouse in Method I and Method II.

**Additional file 5: Table S5.** Genetic diversity of each locus in miracidia from the liver/stool of mice in Method I and Method II.

## Data Availability

The genotypic dataset is available at DRYAD: https://doi.org/10.5061/dryad.s0d566c. Other data are presented in the article and its additional files.
