# Supplementary material for: The genetic variation of different developmental stages of Schistosoma japonicum: do the distribution in snails and pairing preference benefit the transmission?
Source: Parasit Vectors. 2020 Jul 20;13:360. doi: 10.1186/s13071-020-04240-w (PMC7372819; doi:10.1186/s13071-020-04240-w)
Supplement: Supplementary file 1 — Additional file 1: Table S1. The information for nine microsatellite markers used in this study. [file 13071_2020_4240_MOESM1_ESM.pdf]

Additional file 1: Table S1 The information for nine microsatellite markers used in this study

Name	Repeat motif	Primers (5'-3')	Tm (℃)[a]	GenBank No.	Length (bp)[a]	No. of alleles[a]	*Ho* [a]	*He* [a]	Reference	Dye
**Sjp14**	TAA	F: AAATTAACGCACGGACATCA<br>R: AGAATATTGGGACCGGATCA	63	EU262617	206-266	20	0.78-1.00	/	Yin et al., 2008	FAM
**Sj-N127**	ATA	F: ATTTCCCAGTTGTCCAGTTCAG<br>R: TTAGCGAGATTGTTGTTTTACGG	62	JX494378	289-358	28	0.765	0.911	in this study[b]	FAM
**SjP60**	TAT	F: CGATTCATTCATAGCCTGACT<br>R: GAATCCCATCACAGATTAACG	55	AB604245	134-165	10	0.90	0.867	Xiao et al., 2011	HEX
**SjP4**	TAA	F: ACAAGCTCCAATCGTCTCTGA<br>R: GAATACTGCCGCCCTTGTAA	55	EU262607	182-244	14	0.789	0.831	Xiao et al., 2011; Yin et al.,	HEX
**SjP18**	TGA	F: TCCTTTATCTGGGCTGTGGA<br>R: TTTCAGCAGGATAACATGACG	55	AB604199	261-298	7	0.684	0.703	Xiao et al., 2011	HEX
**SjP22**	TTA	F: CAAAGCCTAAACGTCATAGACAG<br>R: CAACCACCGATAAGTAGAGTGGA	55	AB604201	105-167	11	0.85	0.892	Xiao et al., 2011	TRAMA
**Sjp1**	TAA	F: TGAGCACAACTGTATATCCCAAA<br>R: TGGGCAGACATACCAGGTTC	55	EU262604	233-284	17	0.69-1.00	/	Yin et al., 2008	TRAMA
**SjP32**	TTA	F: TGTCACCGAGTCTTCATTAGC<br>R: ACAGTCAGTAGACCTGGATAAAC	55	AB604205	142-192	14	0.95	0.929	Xiao et al., 2011	ROX
**Sjp6**	TAA	F: CGCTATTTATTACTCGGCGTTC<br>R: CGGTCACCAACTCCAAGAAG	55	EU262609	211-298	25	0.72-1.00	/	Yin et al., 2008	ROX

[a] Which was described in related references

[b] This microsatellite was developed in the author's lab
