# Supplementary material for: The genetic variation of different developmental stages of Schistosoma japonicum: do the distribution in snails and pairing preference benefit the transmission?
Source: Parasit Vectors. 2020 Jul 20;13:360. doi: 10.1186/s13071-020-04240-w (PMC7372819; doi:10.1186/s13071-020-04240-w)
Supplement: Supplementary file 2 — Additional file 2: Table S2. Genetic diversity of nine microsatellite loci in all genotyped cercariae from snails of two groups. [file 13071_2020_4240_MOESM2_ESM.pdf]

Additional file 2: Table S2 Genetic diversity of nine microsatellite loci in all genotyped cercariae from snails of two groups

Locus	Group A				Group B			
	Na	***Ae***	***Ar****	***Hs***	***Na***	***Ae***	***Ar***	***Hs***
**Sjp14**	19	8.6	19	0.89	14	6.8	13.8	0.86
**Sj-N127**	19	12.9	19	0.93	19	13.4	19.0	0.93
**SjP60**	13	8.8	13	0.89	12	7.8	11.8	0.88
**SjP4**	17	5.0	17	0.80	15	4.2	14.8	0.76
**SjP18**	13	7.4	13	0.87	13	3.6	13.0	0.73
**SjP22**	22	8.9	22	0.89	20	10.5	19.9	0.91
**Sjp1**	19	10.1	19	0.91	19	7.6	18.8	0.87
**SjP32**	16	10.6	16	0.91	15	9.5	15.0	0.90
**Sjp6**	19	12.6	19	0.92	23	11.5	22.6	0.92
**Mean±SD**	17±2.83	9.4±2.33	17±2.83	0.89±0.04	16.7±3.50	8.3±3.06	16.5±3.45	0.86±0.07

* The number of cercariae from Group A is less than that from Group B, it was used as the sample size in *Ar* test.

Mean±SD: Mean±Standard deviation
