# Supplementary material for: The genetic variation of different developmental stages of Schistosoma japonicum: do the distribution in snails and pairing preference benefit the transmission?
Source: Parasit Vectors. 2020 Jul 20;13:360. doi: 10.1186/s13071-020-04240-w (PMC7372819; doi:10.1186/s13071-020-04240-w)
Supplement: Supplementary file 3 — Additional file 3: Table S3. Genetic diversity of each locus in adult worms from mice infected with two methods. [file 13071_2020_4240_MOESM3_ESM.pdf]

Additional file 3: Table S3 Genetic diversity of each locus in adult worms from mice infected with two methods

Locus	Method I (357 worms)				Method II (344 worms)			
	Na	*Ae*	*Ar*	*Hs*	*Na*	*Ae*	*Ar**	*Hs*
**Sjp14**	17	5.5	17.0	0.82	15	6.2	14.9	0.84
**Sj-N127**	17	7.8	17.0	0.87	19	10.6	19.0	0.91
**SjP60**	13	7.2	12.9	0.86	11	7	11.0	0.86
**SjP4**	13	3.0	12.9	0.66	16	2.9	16.0	0.65
**SjP18**	12	6.0	12.0	0.84	13	4.8	13.0	0.79
**SjP22**	20	6.7	19.7	0.85	24	9	24.0	0.89
**Sjp1**	17	10.0	17.0	0.90	21	10.6	21.0	0.91
**SjP32**	14	8.6	14.0	0.89	15	7.7	15.0	0.87
**Sjp6**	20	11.7	19.9	0.92	22	11	21.9	0.91
**Mean±SD**	16.0±2.85	7.4±2.41	16.0±2.81	0.85±0.07	17.0±4.14	7.8±2.66	17.0±4.14	0.85±0.08

* The number of worms from Method I is less than that from Method II, it was used as the sample size in *Ar* test.

Mean±SD: Mean±Standard deviation
