# Supplementary material for: The genetic variation of different developmental stages of Schistosoma japonicum: do the distribution in snails and pairing preference benefit the transmission?
Source: Parasit Vectors. 2020 Jul 20;13:360. doi: 10.1186/s13071-020-04240-w (PMC7372819; doi:10.1186/s13071-020-04240-w)
Supplement: Supplementary file 4 — Additional file 4: Table S4. Genetic diversity of combined nine loci in worms from each mouse in Method I and Method II. [file 13071_2020_4240_MOESM4_ESM.pdf]

Additional file 4: Table S4 Genetic diversity of combined nine loci in worms from each mouse in Method I and Method II

ID of mouse	Method I				ID of mouse	Method II			
	Na	***Ae***	***Ar***	***Hs***		***Na***	***Ae***	***Ar***	***Hs***
M1	9.6±1.5	6.1±1.8	6.6±0.9	0.85±0.09	M1	10.7±2.1	6.6±2.4	6.9±1.1	0.85±0.08
M2	9.1±1.6	5.7±1.9	6.2±1.0	0.83±0.07	M2	10.2±2.3	6.6±2.4	6.9±1.3	0.85±0.1
M3	7.7±1.7	5.3±1.5	5.9±1.0	0.84±0.07	M3	11.3±2.2	6.4±2.3	6.7±1.1	0.83±0.1
M4	6.9±1.3	5.5±1.7	6.2±1.1	0.86±0.07	M4	10.0±2.1	6.3±2.2	6.7±1.2	0.84±0.11
M5	6.8±1.5	5.0±1.5	6.0±1.2	0.84±0.10	M5	10.3±2.6	7.3±2.4	7.3±1.4	0.87±0.1
M6	8.2±1.7	5.4±1.8	6.4±1.1	0.83±0.09	M6	9.8±1.7	6.3±1.6	6.8±0.8	0.85±0.08
M7	8.7±2.1	5.4±1.7	6.1±1.2	0.81±0.12	M7	11.6±2.5	7.6±2.5	7.4±1.1	0.88±0.06
M8	7.6±1.7	4.8±1.6	5.6±1.0	0.80±0.09	M8	10.3±1.4	6.8±2.0	7.0±0.9	0.87±0.06
M9	8.1±1.6	6.2±1.7	6.5±1.0	0.87±0.05	M9	6.6±1.0	5.2±1.3	6.6±1.0*	0.87±0.07
M10	8.3±1.6	5.5±2.2	6.3±1.1	0.82±0.09	M10	9.6±1.6	5.9±1.6	7.1±1.0	0.86±0.07
M11	7.4±1.4	5.2±1.7	5.9±1.0	0.82±0.08	M11	9.6±1.0	6.3±1.5	6.7±0.7	0.86±0.06
M12	6.8±1.8	4.5±1.3	5.7±1.2	0.82±0.09	M12	11.1±2.6	6.8±2.6	7.0±1.1	0.86±0.08
M13	9.4±1.3	5.9±1.7	6.6±0.8	0.85±0.05	M13	10.0±2.2	6.7±2.3	6.9±1.1	0.86±0.08
M14	9.0±2.2	6.0±2.0	6.6±1.3	0.85±0.07	M14	10.7±1.5	6.0±1.5	6.8±0.7	0.85±0.05
M15	9.1±1.9	7.0±2.1	7.1±1.2	0.88±0.09	M15	10.2±2.1	6.3±2.3	6.7±1.0	0.84±0.08
M16	7.8±1.6	5.8±1.7	6.7±1.2	0.86±0.09	M16	11.6±2.5	7.1±2.4	6.6±1.0	0.85±0.09
M17	8.1±1.8	6.4±1.8	6.6±1.1	0.87±0.04	M17	10.1±1.9	6.4±2.3	6.8±1.1	0.85±0.09
M18	10.9±2.1	6.4±2.0	6.5±1.1	0.84±0.09	M18	9.7±2.1	6.5±2.1	6.8±1.1	0.86±0.07
M19	7.4±1.5	5.5±1.5	6.6±1.1	0.86±0.05	M19	9.9±1.6	6.5±1.3	6.9±0.7	0.87±0.04
M20	7.9±1.6	6.2±1.8	6.4±1.2	0.85±0.12	M20	10.2±1.9	6.2±1.9	6.8±1.0	0.85±0.09
M21	8.9±1.4	5.9±1.7	6.6±0.9	0.85±0.06	M21	11.9±2.2	5.6±1.5	6.1±0.8	0.82±0.06
M22	6.4±1.0	4.7±0.9	6.0±0.9*	0.85±0.04	M22	10.1±1.4	6.5±2.4	6.8±1.0	0.85±0.08
M23	10.7±1.9	7.0±2.2	7.3±1.0	0.88±0.08					
M24	9.7±1.2	6.9±1.8	7.3±0.8	0.89±0.07					
M25	9.4±1.9	6.5±2.1	6.8±1.1	0.87±0.07					
M26	8.3±1.3	4.8±1.1	6.0±0.9	0.82±0.06					
M27	8.7±1.7	6.1±1.8	6.4±0.9	0.86±0.06					
M28	7.9±1.9	5.6±1.6	6.0±1.0	0.84±0.06					
M29	8.7±1.0	5.6±1.8	6.3±0.8	0.84±0.08					
M30	10.2±2.2	6.4±2.5	6.7±1.3	0.84±0.09					
M31	7.3±1.0	5.4±1.4	6.4±0.8	0.85±0.07					

All data are represented in Mean±SD (Mean±Standard deviation)

* The number of worms in this mouse is the least among all mice, it was used as the sample size in *Ar* test.
