# Supplementary material for: The genetic variation of different developmental stages of Schistosoma japonicum: do the distribution in snails and pairing preference benefit the transmission?
Source: Parasit Vectors. 2020 Jul 20;13:360. doi: 10.1186/s13071-020-04240-w (PMC7372819; doi:10.1186/s13071-020-04240-w)
Supplement: Supplementary file 5 — Additional file 5: Table S5. Genetic diversity of each locus in miracidia from the liver/stool of mice in Method I and Method II. [file 13071_2020_4240_MOESM5_ESM.pdf]

Additional file 5: Table S5 Genetic diversity of each locus in miracidia from the liver/stool of mice in Method I and Method II

Locus	Method I									
	Na		***Ae***		***Ar***		***Hs***		***Na***	
	liver	stool	liver	stool	liver*	stool	liver	stool	liver	stool
**Sjp14**	17	15	7.3	7.0	17	14.9	0.87	0.86	12	14
**Sj-N127**	18	19	9.4	10.6	18	18.4	0.90	0.91	18	18
**SjP60**	14	13	5.3	7.8	14	12.7	0.81	0.88	11	12
**SjP4**	13	15	2.9	3.4	13	14.8	0.65	0.71	14	13
**SjP18**	12	10	3.4	4.6	12	9.9	0.71	0.79	14	11
**SjP22**	29	28	6.3	8.5	29	26.6	0.85	0.89	20	18
**Sjp1**	15	21	6.6	9.1	15	19.4	0.85	0.89	20	18
**SjP32**	13	15	4.4	6.8	13	14.6	0.78	0.86	16	15
**Sjp6**	18	17	10.9	12.4	18	16.5	0.91	0.92	19	18
**Mean±SD**	17.0±4.88	17.0±4.92	6.3±2.50	7.8±2.63	17.0±4.88	16.4±4.48	0.81±0.08	0.86±0.06	16.0± 3.23	15.0± 2.70

* The number of miracidia in liver is less than (Method I) or equal to (Method II) that in stool, it was used as the sample size in *Ar* test.
Mean±SD: Mean±Standard deviation

Method II					
Ae		***Ar***		***Hs***	
liver	stool	liver*	stool*	liver	stool
5.8	6.0	12	14	0.83	0.84
12.4	10.9	18	18	0.93	0.91
7.8	7.3	11	12	0.88	0.87
3.3	3.7	14	13	0.70	0.73
4.4	4.9	14	11	0.78	0.80
8.4	6.8	20	18	0.88	0.86
10.3	12.1	20	18	0.91	0.92
7.7	7.5	16	15	0.88	0.87
8.9	8.0	19	18	0.89	0.88
7.7±2.68	7.5±2.50	16.0±3.23	15±2.70	0.85±0.07	0.85±0.06
